# M. Ballin's Case of Abstinence

**Published:** 1806-06

**Authors:** R. Hall

**Affiliations:** Clement's Inn


### *To the Editors of the Medical and Physical Journal.*

GENTLEMEN,

THE following case of voluntary abstinence is of as long duration as probably any other recorded in the annals of medicine. In this instance, as in a similar one related by Dr. Willan, in the Medical Communications, vol. 2d. the conduct of the patient, in imposing upon himself such extraordinary privations, appears to have originated from erroneous notions of religion, or, to speak more correctly, from deranged intellects. To the present case is subjoined a history of the appearances that presented themselves on dissection.

It was drawn up under the inspection of M. Desgenettes, chief physician to the army, physician to the military hospital, and professor of the medical college in Paris; by A Ballin, supernumerary surgeon to the military hospital in Paris. I am, &c.

*Clement's Inn, May* 10 1806. R. HALL.

---

Pierre Landart, 30 years of age, a native of Raucourt in the district of Ardennes, a soldier in the 9th light demi-brigade, was admitted into the military hospital at Paris, the 16th of January 1802. His ticket of admission contained a request from his captain, that the physician would attentively examine the patient, as he affirmed that he took no nourishment whatever.

In consequence of this intimation, Pierre Landart was watched with the greatest strictness. He complained of no disease whatever. The only motive, he asserted, which had induced his officers to send him to the hospital, was his obstinate refusal to swallow food of any kind, in which he had persisted for two years.

My

My first endeavour was to learn the cause of this man's melancholy, who remained the greatest part of the day in a reclining posture, with his head resting on the hand and arm of the right side. With this intention, I put several questions to him, to all of which he replied vaguely, and without giving me the details I solicited. Conceiving the presence of his comrades might embarrass him, I proposed retiring, to which he readily assented; and when alone, related to me, without hesitation, the history of his disease.

He had been originally bred to the trade of a bricklayer, had received little education, and lived like other men in a similar station of life. Three years previous to this attack, he had been subject to no indispositon whatever; when suddenly becoming melancholy, he grew disgusted with the society of his companions, longed for solitude, and retired to his chamber, where he employed himself very assiduously in reading pious books, such as the Bible, Lives of the Saints, &c. This disposition daily increased, and he partly abandoned his labour, and even abridged his hours of repose, in order that he might read and meditate more constantly. He neither, however, frequented the churches, nor maintained any communication with priests, who, from his own account, had never inspired him with much confidence.

He continued in this frame of mind for about a twelve month, when, on the festival of St. John, after having read and meditated longer than usual, he retired to rest. Scarcely had he fallen asleep, when an angel appeared, and announced to him, that God, satisfied with his prayers and meditations, had chosen him to give to man an example of his power, and commanded him to fast forty days and nights. Filled with gratitude, and proud of the choice of the Divinity, he began from this period to impose on himself the greatest privations; he ate and drank only after long intervals, and when compelled to it by imperious necessity. During this fast of forty days, he grew very thin, and his strength became so much impaired, as to render him unable any longer to perform his accustomed labour. His mother and brother, at whose house he lodged, could not prevail on him to take a greater quantity of food, and every day expected to behold him perish with hunger. At the expiration of the forty days, he beheld in a second dream, the angel who had before appeared to him. This minister of God commended him for the punctuality with which he had fulfilled his former command, and informed him that he was now dead to flesh and blood (these were his own expressions) and that, henceforth, he would be under no necessity of taking any nourishment to support his existence. He then presented him with a vase, which he held in his hand, saying, that the liquor it contained would suffice to support life, and would never become exhausted; at the same time putting the cup to his lips, he filled his mouth with a red liquor of a delicious taste, and an exquisitely agreeable odour; after which he disappeared.

On awakening from his slumbers, Landart still felt, on his lips, and in his mouth, the divine beverage that had been given him, and imagined he possessed the power of renewing it, at will, by the action of suction. Henceforward, he refused all kinds of food; and when, on being much importuned by his relations, he consented to take some slight nourishment, it was almost instantly rejected. Persuaded that he had been thoroughly regenerated, and full of confidence in the Deity, he was contented and happy, and even became somewhat less meagre.

Landart, until this period, remained perfectly unknown in his own province; but he now began to attract considerable attention. It was supposed by many, that he played such an extraordinary part with the view of exempting himself from the military requisition; in consequence of which, he was sent to Paris, and placed in a battalion, in which he remained several months, uniformly refusing every kind of sustenance: eventually, it was determined to send him to the hospital.

Such is the history of this malady, as communicated to me by the patient himself. This man was of the middle size, had a dark complexion, large veins, and a broad chest; he was of a rigid temperament, had black sparkling eyes, an intent look, very black hair, and an extremely fœtid breath; his tongue was very white, his mouth unusually clammy; he exercised continually the motion of suction, and swallowed the saliva he procured, in great quantity, by this means. His pulse was small and feeble, and beat from fifty to sixty in a minute. He slept very little during the night, and never in the day-time; his mouth became dry and parched, while asleep, or after speaking for a short time.

Such food and drink as appeared proper to one in his condition, were presented him; but he uniformly refused every thing, and each morning we found at the head of his bed the food which had been given him on the preceding evening. The reiterated solicitations of his attendants could not overcome his resolution; he remained until until the 26th of March, without taking the least nourishment, either solid or fluid; he, notwithstanding, voided every night an ounce and a half or two ounces of urine, which, on being analysed, displayed no difference from healthy urine, except in containing a greater quantity of acid.

March 26th. He consented to take some drink, and made choice of wine and water, in the proportion of a third of wine to two thirds of water. A pint of this mixture was presented to him, but scarcely had he swallowed half a glassful before he rejected it. I carefully collected what was thus rejected, and found that he had retained about a third of the quantity originally swallowed. This liquid had remained too short a time in the stomach to undergo any change; and, in fact, it was found to be exactly in the same state in which it had been swallowed, with the exception of a small quantity of mucous matter which floated on its surface.

27th. He took two pints of the same beverage, which was rejected, as on the preceding day. The urine was augmented to double its former quantity, and yielded a greater quantity of water when subjected to distillation.

28th. He indicated no desire for any thing, but a little sugar, which he allowed to dissolve in his mouth, during the night; this, he said, enabled him to procure a much greater quantity of the heavenly beverage, which supported his existence. Henceforward, I allowed him every day two ounces of sugar.

29th. He had this day a pint of whey, two thirds of which were rejected.

30th. Having requested a little milk, I indulged him with a pint sweetened with sugar, which he swallowed occasionally in small quantities. Three fourths of it were immediately thrown up; at first, the caseous matter only was rejected, but afterwards the serous part also.

31st. On this day he expressed a wish that the milk might be given to him cold, and without the addition of sugar, in which he was readily indulged. Of the pint thus given to him he rejected the same quantity as before, and in the same state of decomposition.

April 1st. Wishing to accustom him to more solid food, I ordered him three ounces of rice with milk, which he retained about an hour, and then threw up only half of it.

2d. On presenting him with a little panada, he ate it with an appearance of appetite, and retained about one half of it. His urine was less in quantity, for instead of four ounces which he had daily voided since the 27th of March,

March, I collected but three ounces; it was of a deep yellow colour, and presented a very abundant precipitate of uric acid.

3d. He informed me, that he found himself fatigued by the incessant vomitings with which he had been harassed for several days past, and declared a resolution to take nothing farther; in consequence of which, I ordered the attendants not to present him with any food whatever. He also complained of much languor, and requested his dismission.

4th. No food was given him. He had a stool for the first time since his entrance into the hospital, the fæces were hard, dry, brownish, and in small quantity, and the patient experienced much pain in voiding them.

5th. Had no food. The quantity of urine was reduced to two ounces.

6th. Abstinence from food the same as on the three former days. Urine in the same quantity as yesterday. Languid, melancholy, and anxious to leave the hospital.

7th. A day of complete abstinence from both food and drink. Equally anxious as yesterday to obtain his dismission, in the hope of returning to his family. The physician at length yielded to his reiterated applications on this subject, and fixed the following day for his leaving the house.

8th. Displayed the most lively satisfaction, and although weak and feeble, he set out on foot to return to his family.

22d. Pierre Landart, whom we supposed to be far on his way from Paris, was this day brought back on a litter to the hospital. He had only been at the barracks, with his fellow-soldiers, in Oursine-street. After he was put into bed, I approached, and interrogated him, but he had entirely lost his senses. His ideas were perfectly incoherent, and he did not recollect me. I could not discover what had been his conduct since leaving the hospital, whether he had taken any food, or, in short, what had been the circumstances which reduced him to his present situation, so different from that in which he was on leaving the hospital. During his delirium, his religious ideas recurred with redoubled force. He felt, he said, the hand of God weighing heavy upon him. He constantly saw the devil by his side; and unceasingly pursued by him, he refused all kinds of nourishment, and anxiously longed for death.

As he was extremely feeble, we endeavoured to make him swallow some spoonfulls of rice milk; but the muscles cles of the pharynx were in a paralytic state, so that he could get down only a few drops. He, besides, experienced so much pain in the act of deglutition, that he repelled every thing which was held to his lips, and forcibly kept his teeth shut, in order to prevent the introduction of any food into his mouth. Notwithstanding every obstacle, we at last succeeded in getting down a few drops of some liquid, which was almost instantly rejected.

We had next recourse to injections of broth; the four first were retained, but all those administered afterwards were immediately thrown back.

He died at mid-day, on the 26th.

On inspecting the dead body, the following appearances were observed. The brain was of the natural consistence, firmness, and colour. Some whitish concretions were remarked on the superior and internal part of each hemisphere; but there was no effusion of fluid either between the membranes or in the ventricles. The cerebellum was also in a natural state. The vascular system of the head and sinusses were in no respect preternaturally full of blood. The mouth and tongue were completely dry; this last was besides indurated and shrivelled. The parotid, as well as the maxillary and all the salivary glands, were diminished in size; the excretory ducts of these glands were very visible. The pharynx, the larynx, and the aspera arteria were loaded with a very great quantity of mucus. The lungs were uniformly sound, and without any adhesion to the contiguous parts. A slight effusion had taken place into the pericardium. The heart was much loaded with fat, and of a very deep yellow colour. The diaphragm was perfectly sound. The parietes of the abdomen were very much collapsed, and in contact with the vertebral column. The stomach was reduced to a fourth of its usual size, and contained a very great quantity of yellowish mucus. The œsophagus, as well as the cardia and pylorus were sound. The coats of the stomach were very much thickened, hard, and almost in a cartilaginous state. The duodenum, and the other small intestines were filled with bile of a very deep green colour. Their coats were unusually thickened, but not so much as those of the stomach. The transverse colon, in place of running along from the anterior surface of the stomach, described an opposite arch, the concavity of which was turned towards the great border of the stomach. The rectum contained only a very little fæculent matter, and that nearly in a fluid state. The coats of the intestinal canal were very much much thickened, throughout its whole extent. The mesenteric glands exhibited no appearance of any obstruction. The liver was of the usual size, colour and consistence. The venous vessels of the abdomen, and particularly the vena portarum, were full of extremely black blood. The gall bladder was enlarged, and distended with very black, thick, and viscid bile, which exhibited signs of incipient concretion. The pancreas and the spleen were uniformly sound. The omentum was very bulky, and interlarded with a great deal of fat. The kidneys and ureters were in a natural state. The urinary bladder was very small, and its membranes exhibited a still greater degree of thickening than those of the stomach and the intestinal canal.

